# Prognosis of fasting in patients with cerebral venous thrombosis using oral contraceptives

**Published:** 2019-04-04

**Authors:** Masoud Ghiasian, Maryam Mansour, Nasrin Moradian

**Affiliations:** 1 Department of Neurology, Sina Hospital, Hamadan University of Medical Sciences, Hamadan, Iran; 2 Department of Neurology, School of Medicine, Kermanshah University of Medical Sciences, Kermanshah, Iran

**Keywords:** Cranial Sinus Thrombosis, Venous Thrombosis, Oral Contraceptives, Fasting

## Abstract

**Background:** There have been studies that showed a higher incidence of cerebral venous thrombosis (CVT) in Ramadan, a month in which people fast in Muslim countries, which was associated with increasing use of oral contraceptives (OCPs) in women. We aimed to evaluate the effect and prognosis of fasting in patients with CVT using OCPs.

**Methods:** Consecutive patients with diagnosis of CVT in Sina hospital, Hamadan, West of Iran, from May of 2009 to June of 2016 were evaluated, and women using OCPs were included. Other risk factors except fasting were excluded. Clinical presentation and outcomes of CVT was assessed. Patients were followed up for 12 months.

**Results:** 58 patients were included in this study. 31 of these patients had fasting simultaneously. Fasting in patients using OCPs caused significantly higher focal neurological deficit (64.5%, P = 0.018), and higher hemorrhage (66.7%, P = 0.042). At discharge, 51.6% and after three months, 25.8% of patients with fasting had disability [6 > modified Rankin Scale (mRS) >1]. In patients who used OCPs as sole risk factor, 25.9% at discharge and 11.1% after three months had disability.

**Conclusion:** Fasting in patients with CVT using OCPs causes significant increase in focal neurological deficit and hemorrhage, which also increases the hospital stay and lengthens recovery. However, long-term prognosis and mortality of CVT is similar between the two groups.

## Introduction

Cerebral venous sinuses thrombosis (CVT) is an uncommon cerebrovascular disorder.^1^ One of the most important risk factor in women is oral contraceptives (OCPs) which can increase the odds of CVT by 5 to 22 folds.^2^ Women in Islamic countries use OCPs to delay their menstruation, because menstruating women cannot fast during Ramadan.

Ramadan fasting can cause dehydration in individuals which can be associated with increased risk of CVT, particularly in women taking OCPs.^3^

Our objective was to evaluate the effect and prognosis of fasting in patients with CVT using OCPs.

## Materials and Methods

This analytical cross-sectional study was conducted from May of 2009 to June of 2016 in Sina hospital, Hamadan, West of Iran. 

Patients with CVT using OCPs were included in this study. Patients with risk factors other than OCP consumption or fasting were excluded. All participants gave their consent. 

Follow up of patients was done by face-to-face interview. Disability was recorded according to the modified Rankin Scale (mRS).

Data was analyzed using SPSS software (version 20, IBM Corporation, Armonk, NY, USA). P-value < 0.05 was considered significant.

## Results

123 women were diagnosed with CVT in the study period. 67 women were using OCPs. 9 of these patients had other risk factors as well as OCP, and were excluded from the study. Overall, 58 patients with mean age of 38.91 ± 8.39 years were included. 31 of these patients had fasting simultaneously. None of the patients had fasting as a sole risk factor. Mean hospital stay was 13.52 ± 14.73 days for patients with fasting and 9.62 ± 2.54 days for patients without fasting.

In patients with fasting, focal neurological deficit (64.5% versus 33.3%, P = 0.018) and hemorrhagic infarction (66.7% versus 30.8%, P = 0.042) was significantly higher. In these patients mean hospital stay was 19.58 ± 22.76 days.

All of the patients were followed up for at least 12 months. Outcomes for death and disability are presented in table 1.

There was no significant difference in mortality rate in patients with fasting and those without fasting at discharge (P = 0.473) and at the end of follow-up (P = 0.886).

At discharge, 66.7% of patients without fasting and 45.2% of those with fasting had no significant disability (P = 0.100). 

At the end of follow-up period, 81.5% of patients without fasting and 80.6% of those with fasting had no significant disability (P = 0.935).

## Discussion

In previous articles, it was reported that the incidence of CVT was higher in Islamic countries in Ramadan.^3^ Saeidi, et al. reported in Iran that incidence of CVT were significantly higher in Ramadan than other months.^4^

In this study, we compared patients using CVT using OCPs with fasting to those using OCPs alone. 

Fasting in patients using OCPs significantly increased focal neurologic deficits and hemorrhagic infarction at presentation. As a result, in patients with fasting, disability was insignificantly higher than those without fasting at discharge (51.6% versus 25.9%). 

Mortality rate in our study was similar to other studies in Iran (13.3%).^5^ However, it was slightly higher than the mortality rate in women with gender-specific risk factors in western countries (4.0%).^6^

At 6-months follow-up, 80.6% of patients with fasting and 81.5% of patients with OCPs, as sole risk factor, had complete recovery which was similar to 85.0% of women with gender-specific risk factors of International Study on Cerebral Vein and Dural Sinus Thrombosis (ISCVT).^6^

Our study was limited in the number of patients included. We did not compare long-term versus short-term use of OCPs.

**Table 1:** Outcome of patient

**mRS**	**Outcome at discharge**		**P**	**Outcome at 12 months**		**P**
	**Patients with only ** **OCP (n = 27)**	**Patients with OCP ** **and fasting (n = 31)**		**Patients with only ** **OCP (n = 27)**	**Patients with OCP and ** **fasting (n = 31)**	
	**n (%)**	**n (%)**		**n (%)**	**n (%)**	
0	1 (3.7)	0 (0)	0.381	21 (77.8)	20 (64.5)	0.463
1	17 (63.0)	14 (45.2)		1 (3.7)	5 (16.1)	
2	4 (14.8)	9 (29.0)		3 (11.1)	4 (12.9)	
3	3 (11.1)	6 (19.4)		0 (0)	0 (0)	
4	0 (0)	0 (0)		0 (0)	0 (0)	
5	0 (0)	1 (3.2)		0 (0)	0 (0)	
Deaths	2 (7.4)	1 (3.2)		2 (7.4)	2 (6.5)	

mRS: Modified ranking scale; OCP: Oral contraceptives

## Conclusion

Fasting in patients with CVT using OCPs causes significant increase in focal neurological deficit and hemorrhagic infarction at presentation, which also increases the hospital stay. However, it does not significantly change long-term prognosis and mortality of CVT in women using OCPs.

## Notes:

**How to cite this article:** Ghiasian M, Mansour M, Moradian N. Prognosis of fasting in patients with cerebral venous thrombosis using oral contraceptives. Iran J Neurol 2019; 18(2): 82-4.
